# The influence of rurality on melanoma diagnosis in Indiana: A retrospective cohort study

**DOI:** 10.1002/cnr2.2072

**Published:** 2024-04-10

**Authors:** Gloria R. Xue, Marie Clark, Claudia Morr, James E. Slaven, Syril Keena T. Que

**Affiliations:** ^1^ Department of Dermatology Indiana University School of Medicine Indianapolis Indiana USA; ^2^ Department of Biostatistics and Health Data Science Indiana University School of Medicine Indianapolis Indiana USA

**Keywords:** cutaneous melanoma, Indiana, melanoma diagnosis, rural disparities, rural health

## Abstract

**Background:**

Research from across the United States has shown that rurality is associated with worse melanoma outcomes. In Indiana, nearly a quarter of all residents live in rural counties and an estimated 2180 cases of melanoma will be diagnosed in 2023.

**Aims:**

This study examines how geographical location affects the stage of melanoma diagnosis in Indiana, aiming to identify and address rural health disparities to ultimately ensure equitable care.

**Methods and Results:**

Demographics and disease characteristics of patients diagnosed with melanoma at Indiana University Health from January 2017 to September 2022 were compared using Students *t*‐tests, Wilcoxon tests, chi‐squared or Fisher's exact tests. Patients from rural areas presented with more pathological stage T3 melanomas (15.0% vs. 3.5%, *p* < 0.001) in contrast to their urban counterparts. Additionally, rural patients presented with fewer clinical stage I melanomas (80.8% vs. 89.3%) and more clinical stage II melanomas (19.2% vs. 8.1%), compared to urban patients, with no stage III (*p* = 0.028). Concerningly, a significantly higher percentage of the rural group (40.7%) had a personal history of BCC compared to the urban group (22.6%) (*p* = 0.005) and fewer rural patients (78.0%) compared to urban patients (89.4%) received surgical treatment (*p* = 0.016).

**Conclusion:**

Patients from rural counties in Indiana have higher pathological and clinical stage melanoma at diagnosis compared to patients from urban counties. Additionally fewer rural patients receive surgical treatment and may be at higher risk of developing subsequent melanomas.

## 1 INTRODUCTION

Research has shown that rurality is associated with higher age‐adjusted melanoma incidence rate and melanoma‐specific mortality rates, particularly in men.^1^, ^2^, ^3^ Twenty‐two percent of Indiana residents live in counties with rural designations, and it is estimated that 2180 cases of melanoma will be diagnosed in Indiana in 2023.^4^, ^5^ To establish equitable care, it is imperative to identify and address associations between rurality and melanoma disease progression. This study explores the impact of geographical location on melanoma disease characteristics at diagnosis in Indiana.

## 2 METHODS

A retrospective review of 347 patients diagnosed with melanoma at Indiana University Health between January 2017 and September 2022 was conducted. Indiana University Health is the largest healthcare system in Indiana consisting of 16 hospitals and over 200 locations statewide. Data obtained included address, age, gender, and tumor characteristics at diagnosis. Patients were designated according to U.S. Census definitions: “urban” if they resided in a county where the population was ≥50 000 or “rural” if they resided in a county where the population was <500 000. We excluded four patients who resided outside of Indiana. Patient demographics and disease characteristics were compared using Student's *t*‐tests and Wilcoxon tests for continuous variables, and chi‐squared tests (Fisher's Exact when over 20% of cells had an expected value <5) for categorical variables. A *p*‐value cutoff of 0.05 was determined to be significant. All analytic assumptions were verified. Analyses were performed using SAS v9.4 (SAS Institute, Cary, NC).

## 3 RESULTS

Of the 343 patients included in the analysis, 59 (17.2%) and 284 (82.3%) resided in rural and urban counties, respectively. Average age at diagnosis was similar across both groups at 64.9 years old for rural subjects and 61.8 for urban subjects (*p* = 0.200). Additionally, there were no significant differences in gender, race, ethnicity, insurance status, or veteran status (Table 1).

**TABLE 1:** “Demographics and past medical history of rural and urban patients with melanoma in Indiana”.

	Rural (*n* = 59)		Urban (*n* = 284)		*p*‐Value
	*N*	%	*N*	%	
Average age at diagnosis	64.9 (15.8)		61.8 (16.4)		0.200
Gender					
Male	31	56.4	152	55.5	0.904
Female	24	43.6	122	44.5	
Race					
White	54	100	262	97.8	0.594
Black	0	0	6	2.2	
Ethnicity					
Hispanic	0	0	3	1.1	0.230
Not Hispanic	54	98.2	270	98.9	
Not reported	1	1.8	0	0	
Insured					
No	0	0	1	<0.01	>0.999
Yes	55	100	268	99.6	
Veteran					
Yes	0	0	3	1.1	0.238
No	54	100	250	94.0	
Unknown	0	0	13	4.9	
Personal history of melanoma					
Yes	16	29.1	55	20.1	0.150
No	39	70.9	218	79.9	
Number of previous melanomas	0 (0–1)		0 (0–0)		0.208
Personal history of SCC					
Yes	10	18.9	60	22.2	0.588
No	43	81.1	210	77.8	
Personal history of BCC					
Yes	22	40.7	61	22.6	0.005
No	32	59.3	209	77.4	
Immunosuppressed					
Yes	3	5.7	7	2.6	0.214
No	50	94.3	264	97.4	
Family history of melanoma					
Yes	3	5.5	28	10.3	0.487
No	45	81.8	206	75.5	
Unknown	7	12.7	39	14.3	
Family history of NMSC					
Yes	3	5.6	29	10.7	0.351
No	33	61.1	172	63.2	
Unknown	18	33.3	71	26.1	
Regular dermatologist					
No	21	38.2	131	47.8	0.361
Yes	32	58.2	136	49.6	
Unknown	2	3.6	7	2.6	
TBSE screening interval at time of diagnosis					
Every 3 months	7	12.7	22	8.0	0.830
Every 6 months	11	0.2	46	16.8	
Every 12 months	8	14.5	39	14.2	
>12 months	6	10.9	34	12.4	
No prior TBSE	21	38.2	117	42.7	
Unknown	2	3.6	16	5.8	

*Note*: Values are means (standard deviations) for age and frequencies (percentages) for categorical variables, with *p*‐values from Student's *t*‐tests and chi‐square tests (Fischer's Exact where necessary), respectively. Other values are medians (IQRs) for continuous variables, with *p*‐values from Wilcoxon tests. Due to missing data, counts from some variables do not add up to the total.

Compared to patients living in urban areas, patients living in rural areas presented with higher stage melanomas at the time of diagnosis. Rural patients presented with fewer pathologic stage T1 melanomas than urban patients (66.1% vs. 78.9%, *p* = 0.035) but more stage T3 melanomas (15.3% vs. 3.5%, *p* < 0.001). In terms of clinical stage, there was significant heterogeneity in proportions (*p* = 0.028), with fewer clinical stage I melanomas among rural patients than urban patients (80.8% vs. 89.3%) and a higher percentage of clinical stage II melanomas from patients from rural areas as compared to those from urban areas (19.2% vs. 8.1%), with no rural patients having clinical stage III disease (Table 2). No statistically significant differences were found in Breslow thickness, Clark level, tumor diameter, and histological subtypes or presence of mitoses, ulceration, and lymphovascular invasion, or lymph node biopsies between the two groups (Table 2).

**TABLE 2:** “Tumor characteristics at diagnosis of rural and urban patients with melanoma in Indiana”.

	Rural (*n* = 59)		Urban (*n* = 284)		*p*‐Value
	*N*	%	*N*	%	
Pathologic stage					
Tx	0	0	0	0	n/a
T0	0	0	0	0	n/a
Tis	0	0	1	0.4	>0.999
T1	39	66.1	224	78.9	0.035
T2	3	5.1	30	10.6	0.233
T3	9	15.3	10	3.5	<0.001
T4	2	3.4	8	2.8	0.684
Clinical stage					
I	42	80.8	243	89.3	0.028
II	10	19.2	22	8.1	
III	0	0	7	2.6	
Breslow thickness	0.5 (0.3–1.0)		0.5 (0.3–0.8)		0.536
Clark level					
II	16	28.1	70	24.9	0.855
III	24	42.1	128	45.6	
IV	17	29.8	83	29.5	
Mitoses					
No	44	77.2	233	82.6	0.333
Yes	13	22.8	49	17.4	
Presence of ulceration					
No	51	89.5	266	94.7	0.139
Yes	6	10.5	15	5.3	
Lymphovascular invasion					
No	55	96.5	272	96.5	>0.999
Yes	2	3.5	10	3.6	
Greatest dimension (cm)	0.9 (0.6–1.2)		0.8 (0.5–1.2)		0.664
Histological subtype					
In situ	0	0	1	0.4	0.104
Superficial spreading	34	65.4	203	75.8	
Nodular	6	11.5	7	2.6	
Lentigo maligna melanoma	7	13.5	25	9.3	
Acral lentiginous	0	0	2	0.8	
Not addressed	0	0	6	2.2	
Other	5	9.6	24	9.0	
Sentinel lymph node biopsy					
Yes	12	25	55	20.7	0.501
No	36	75	211	79.3	
Number of nodes sampled	2 (1–2)		1 (1–3)		0.902
Number of positive nodes	0 (0–0)		0 (0–0)		0.270

*Note*: Values are medians (IQRs) for continuous variables and frequencies (percentages) for categorical variables, with *p*‐values from Wilcoxon tests and chi‐square tests (Fisher's Exact where necessary), respectively. Due to missing data, counts from some variables do not add up to the total.

For the treatment of melanoma, 89.4% of urban patients received surgery compared to 78.0% of rural patients (*p* = 0.016). However, type of surgery and reconstruction type after surgery were not significantly different between the two groups (Table 3). Additionally, little to no rural patients (0%) and urban patients (1.1%) (*p* > 0.999) received immunotherapy.

**TABLE 3:** “Definitive treatment of melanoma for rural and urban patients in Indiana”.

	Rural (*n* = 59)		Urban (*n* = 284)		*p*‐Value
	*N*	%	*N*	%	
Surgery					
Yes	46	78.0	254	89.4	0.016
No	13	22.0	30	10.6	
Type of surgery					
Mohs	2	4.3	17	6.7	0.548
Wide local excision	44	95.7	237	93.3	
Reconstruction type					
Intermediate repair	30	62.5	104	40.8	0.239
Complex repair	7	14.6	55	21.6	
Full thickness graft	3	6.3	21	8.2	
Split thickness graft	0	0	1	0.4	
Random pattern flap	0	0	7	2.8	
Free flap	3	6.3	18	7.1	
Other	3	6.3	20	7.8	
Unknown	2	4.2	29	1.4	
Immunotherapy					
Yes	0	0	3	1.1	>0.999
No	59	100	281	98.9	

*Note*: Values are frequencies (percentages) for categorical variables with *p*‐values from chi‐square tests (Fisher's Exact where necessary).

While immunosuppression status, personal history of melanoma and squamous cell carcinoma (SCC), and number of previous melanomas were not significantly different, a higher percentage of the rural population (40.7%) had a personal history of basal cell carcinoma (BCC) compared to the urban population (22.6%) (*p* = 0.005) (Table 1). Family history of skin cancers including melanoma and nonmelanoma skin cancers (NMSC) were also not different between the two groups (Table 1). A similar percentage of rural and urban patients did not have a regular dermatologist, 38.2% and 47.8%, respectively (*p* = 0.361), or a prior total body skin exam (TBSE), 38.2% and 42.7%, respectively (*p* = 0.839).

## 4 DISCUSSION

Our findings did not identify significant differences in demographics relating to age, race, gender, or ethnicity at melanoma diagnosis; however, our results demonstrate that patients from rural counties in Indiana have higher pathological and clinical stage melanoma at diagnosis compared to patients from urban counties. This observation is consistent with previous investigations that have found rural patients to be diagnosed with late‐stage melanoma more often that urban patients.^6^, ^7^ Concerningly, other studies have found higher stage melanoma at diagnosis to be associated with poorer prognosis and higher mortality.^8^

In terms of treatment, rural patients were significantly less likely to receive surgery than their urban counterparts in Indiana. This conclusion is consistent with nationwide trends that show melanoma patients from rural areas are less likely to receive recommended surgery.^9^ Given that surgery is still the best treatment option for localized, invasive melanoma, which represents the vast majority of tumors found in both rural and urban groups in this study, this finding is especially concerning as it pertains to melanoma‐specific survival.

Furthermore, we also found that a significantly higher percentage of melanoma patients from rural counties had a personal history of BCC. Based on the understanding that ultraviolet radiation (UVR) contributes to the development of both melanomas and BCCs, we believe that the rural group may have experienced more exposure to UVR prior to melanoma diagnosis and thus be at increased risk of subsequent melanoma diagnoses.^10^ This belief is supported by a study that found risk of melanoma to be 6.6 times higher in patients who had a prior BCC compared those who did not.^11^ Thus, there is significant interest in understanding the factors that result in higher stage of melanoma at diagnosis and less surgical treatment in the rural population.

While the objective of this study was not to identify the factors causing disparities in melanoma diagnosis and outcomes in rural and urban populations, our data suggests that insurance status, which impacts access to care, was not the causative factor. This finding may be due to the large number of subjects in both groups of our study who were >65 years old, qualifying them for Medicare in addition to employer‐sponsored insurance. Availability of TBSEs and dermatologist densities are also sometimes noted to influence melanoma outcomes. Studies have shown a decreased all‐cause mortality associated with melanomas diagnosed during TBSE, likely as a result of earlier diagnosis.^12^ While primary care physicians sometimes conduct these exams, TBSEs are most commonly completed by dermatologists and a study from 2018 found that 88% of rural counties do not have a single dermatologist.^13^ It has also been found that greater dermatologist density is associated with lower melanoma mortality.^14^ In our study, similarly large portions of rural and urban subjects did not have a regular dermatologist (urban: 47.8% and rural: 38.2%) and did not have a prior TBSE (urban: 42.7% and rural: 38.2%). Based on these findings, it is assumed that many of these melanomas were not detected as part of a TBSE, but rather based on patient or physician concern. While we are unable to draw conclusions regarding dermatologist density in Indiana, our analysis indicates that more dermatologists are needed throughout the entire state.

Limitations of our study include the restrictive nature of the rural versus urban designation, which defines a person's residence based on a population cutoff without consideration of the heterogeneity of population density, geographic proximity to major medical centers, or density of dermatologists per capita. Additionally, missing data across some of the study variables limits the statistical power of our analysis, particularly for the rural group of patients. Similarly, Fidanzi et al. found that incomplete data in cancer registries underestimated incidence of melanoma.^15^ This highlights the importance of notifying the appropriate national‐ and state‐specific cancer registries of all diagnosed melanoma cases as well as the associated socioeconomic variables, which would prevent underpowered studies in the future and provide more comprehensive data to inform investigations of factors leading to the identified disparities. Given these limitations and the evidence of disparities in this study, future investigations could further refine the definition of rural communities, assess the factors influencing access to care, including dermatologist density and socioeconomic variables, and address gaps in patient knowledge regarding skin cancer recognition and prevention.

## 5 CONCLUSION

Melanoma patients from rural counties in Indiana present with higher pathologic and clinical stage at diagnosis compared to their urban counter parts. Concerningly, these patients are not only less likely to receive surgical treatment but are also at higher risk of developing subsequent melanomas. Our findings highlight the disparities that rural communities in Indiana face regarding melanoma diagnosis and treatment, and pave the way for future studies to identify and address the factors leading to these findings, to ultimately ensure equitable care.

## AUTHOR CONTRIBUTIONS

**Gloria R. Xue:** Conceptualization (lead); data curation (equal); writing – original draft (lead). **Marie Clark:** Writing – review and editing (equal). **Claudia Morr:** Conceptualization (supporting); data curation (equal); project administration (equal). **James E. Slaven:** Formal analysis (equal). **Syril Keena T. Que:** Conceptualization (supporting); funding acquisition (lead); supervision (lead); writing – review and editing (equal).

## CONFLICT OF INTEREST STATEMENT

Dr. Que is an Investigator for Castle Biosciences and has provided consultation to American College of Physicians Dynamed. The authors have stated explicitly that there were no incentives or transactions, financial or otherwise, relevant to this manuscript.

## ETHICS STATEMENT

This study was reviewed and approved by the Indiana University HRPP Institutional Review Board, Indianapolis, IN. Approval #18178. Patient consent was not applicable to this study.

## Data Availability

All the data to support the findings of this study, which are reported in Tables 1 and 2, were calculated by the authors of this study and based on data obtained from Indiana University Health. The participants of this study did not give written consent for their data to be shared publicly. Due to the sensitive nature of the research, this data is not available.
